# Patients’ perception of safety climate in Irish general practice: a cross-sectional study

**DOI:** 10.1186/s12875-021-01603-9

**Published:** 2021-12-27

**Authors:** Caoimhe Madden, Sinéad Lydon, Andrew W. Murphy, Paul O’Connor

**Affiliations:** 1 Department of General Practice, School of Medicine, National University of Ireland Galway, 1 Distillery Road, Lower Newcastle, Galway, Ireland; 2 Irish Centre for Applied Patient Safety and Simulation, Galway University Hospital, Galway, Ireland; 3 School of Medicine, National University of Ireland Galway, Galway, Ireland; 4 HRB Primary Care Clinical Trials Network Ireland, National University of Ireland Galway, Galway, Ireland

**Keywords:** Primary care, General practice, Patient safety, Safety climate, Patient involvement

## Abstract

**Background:**

Although patients have the potential to provide important information on patient safety, considerably fewer patient-report measures of safety climate (SC) have been applied in the primary care setting as compared to secondary care. Our aim was to examine the application of a patient-report measure of safety climate in an Irish population to understand patient perceptions of safety in general practice and identify potential areas for improvement. Specifically, our research questions were:

1. What are patients’ perceptions of SC in Irish general practice?

2. Do patient risk factors impact perceptions of SC?

3. Do patient responses to an open-ended question about safety enhance our understanding of patient safety beyond that obtained from a quantitative measure of SC?

**Methods:**

The Patient Perspective of Safety in General Practice (PPS-GP) survey was distributed to primary care patients in Ireland. The survey consisted of both Likert-response items, and free-text entry questions in relation to the safety of care. A series of five separate hierarchical regressions were used to examine the relationship between a range of patient-related variables and each of the survey subscales. A deductive content analysis approach was used to code the free-text responses.

**Results:**

A total of 584 completed online and paper surveys were received. Respondents generally had positive perceptions of safety across all five SC subscales of the PPS-GP. Regarding patient risk factors, younger age and being of non-Irish nationality were consistently associated with more negative SC perceptions. Analysis of the free-text responses revealed considerably poorer patient perceptions (*n* = 85, 65.4%) of the safety experience in primary care.

**Conclusion:**

Our findings indicate that despite being under-utilised, patients’ perceptions are a valuable source of information for measuring SC, with promising implications for safety improvement in general practice. Further consideration should be given to how best to utilise this data in order to improve safety in primary care.

## Background

Approximately 21.4 million consultations are conducted by General Practitioners (GPs) in Ireland annually [1]. Given that 2–3% of primary care consultations are associated with the occurrence of a Patient Safety Incident [2] (PSI; an event or circumstance that could have resulted, or did result, in unnecessary harm to a patient [3]), there is ample opportunity for safety incidents to occur considering the volume of patient contacts in Irish general practice. Further, there is a growing complexity of primary health care delivery, with the interaction of multiple patient factors including age, multimorbidity, and polypharmacy posing significant and ongoing challenges for GPs [4], having been associated with a higher risk of PSIs in primary care [5–7].

Such data highlight the need for the consistent measurement and monitoring of safety in general practice. The measurement of safety climate (SC), defined as the perceived state of safety in an organisation at a given time and place [8], is recognised as a ‘leading’ [9], or proactive, method of safety measurement in healthcare [10, 11]. SC measures are typically quantitative in design, and examine the perspectives of healthcare professionals (HCPs) [12]. However, it has been suggested that the patient perspective may also constitute a valuable source of information on patient safety [7, 13]. Further, patients have expressed interest in being involved in the safety of their care to improve outcomes [14].

A recent systematic review of patient-report SC measurement in healthcare found that considerably fewer measures have been developed for use in the primary care setting as compared to secondary care [15]. Further, extant patient-report SC measures were found to have limited psychometric properties, usability, and/or did not sufficiently address all of the domains of SC [15]. Such findings culminated in the subsequent development of a novel patient-report measure of SC; the Patient Perspective of Safety in General Practice (PPS-GP), which has demonstrated favourable validity, reliability, and usability [16]. This tool creates the potential to capitalise on patient insights and experiences to improve the safety of primary care delivery.

Considering GPs have cited difficulties in understanding how best to measure and improve patient safety in their practices [17], it may be useful to explore how the PPS-GP can be used to identify patient or practice characteristics that predict perceptions of safety or to consider the areas in which patients may perceive safety to be suboptimal. Further, a focus on high risk patients may improve the safety of care by helping pinpoint areas requiring improvement [18]. Therefore, the aim of this study was to examine the application of a patient-report measure of SC with primary care patients in the Republic of Ireland to understand patient perceptions of safety in general practice and identify potential areas for improvement. Our specific research questions were:

1. What are patients’ perceptions of SC in Irish general practice?
2. Do patient risk factors impact perceptions of SC?
3. Do patient responses to an open-ended question about safety enhance our understanding of patient safety beyond that obtained from a quantitative measure of SC?

## Methods

### Design

The study used a cross-sectional survey design. The study is reported in accordance with the ‘Strengthening the Reporting of Observational Studies in Epidemiology’ (STROBE) Statement [19].

### Measure administered

The first section of the survey presented the PPS-GP (16) which contained 37-items pertaining to SC across five subscales: (1) Feeling of safety with the GP (e.g., ‘*I trust the GP*’; 7 items); (2) Practice staff efficiency and teamwork (e.g., ‘*Staff in the practice help each other*’; 12 items); (3) Staff stress and workload (e.g., ‘*The GP is usually rushed*’; 7 items); (4) Patient knowledge and accountability (e.g., ‘*I know how to report issues with my care*’; 6 items), and; (5) Safety systems and behaviours (e.g., ‘*The instructions I am given about my care are clear*’; 5 items). Items were rated on a 5-point Likert response scale, ranging from ‘1’ (‘strongly disagree’) to ‘5’ (‘strongly agree’). Additionally, there were two free-text entry questions (any additional comments in relation to the safety of care received while visiting the practice, and the opportunity to list areas where changes could be made to GP care [20]), and two global safety items concerned with the participant’s overall safety rating of the practice (scored from 1 to 10) and the likelihood of their recommending the practice to friends and family [21] (five response options, ranging from ‘extremely unlikely’ to ‘extremely likely’). The PPS-GP has demonstrated good internal reliability and content, construct, and convergent validity, and favourable markers of participant usability (e.g., readability and duration of completion). Psychometric properties of the PPS-GP are comprehensively detailed in a previous publication by the research team [16].

The second section of the survey presented questions seeking demographic information including gender, nationality, and whether the respondent was private or publicly funded. The Republic of Ireland, the General Medical Services (GMS) scheme entitles patients with incomes below a threshold on a means tested basis or with specified illnesses to obtain a medical card, which provides free health care in the form of prescriptions and GP visits, while a GP visit card, or doctor visit card (DVC; typically held by individuals over the age of 70 regardless of income and children under the age of six) entitles a patient to free GP access only [22]. Additional questions included ‘proxies’ for identifying high-risk patient factors [23, 24] and included: age; hospital admission within the last 12 months; fall within the last 12 months; frequency of GP attendance within the last 12 months; presence of chronic diseases (e.g., chronic obstructive pulmonary disease, cardiovascular disease, heart failure), and the number of repeat medication items.

### Participant recruitment and data collection

A combination of convenience and purposive sampling were used to recruit participants as part of a previous study; data from the same participant sample were used in the current study [16]. Data were collected between February and June 2020. Inclusion criteria specified that participants were required to be over 18 years old, English-speaking, and to have visited a GP in the Republic of Ireland within the previous 24 months. The study was advertised on social media (e.g., Twitter), to patient advocacy groups, in local newspapers and via interviews with local radio stations. In order to ensure inclusion of ‘high-risk’ patients and members of the public within the sample of respondents, 176 relevant organisations and groups (e.g., community retirement groups, chronic illness support groups) were contacted and asked to share information on the study and/or distribute a copy of the measure to their members [16].

Participants were provided with the choice of completing the survey via paper copy, which was posted to participants alongside a stamped, addressed return envelope, or accessed online via SurveyMonkey, by distributing a survey link to participants’ email addresses. Participation was voluntary, and a prize draw for 50-euro gift vouchers was used to incentivise participation.

### Data analysis

Data were analysed using SPSS (version 26). The items within each of the five subscales were summed to give total subscale scores. Higher scores represented greater perceived SC for each of the subscales, with the exception of *Staff stress and workload*; as six out of seven of these items were negatively worded, the positively worded item was reverse scored such that higher scale scores represented greater perceived stress [16].

#### Patient’s perceptions of safety climate in Irish general practice

Descriptive statistics were used to summarise patients’ perceptions of SC in Irish general practice in relation to each of the five SC subscales, and the two global safety items. As the Kolmogorov-Smirnov test revealed a non-normal distribution of data (*p* < .001), the median for each subscale is reported and the interquartile range as an indicator of dispersion [25].

#### Impact of patient risk factors on safety climate perceptions

A series of five separate hierarchical regressions were used to examine the relationship between a range of patient-related variables and each of the SC subscales, which were treated as the criterion variable in each case. In the regression model, predictors included demographic variables (gender, age, nationality, and GMS status) which were entered in Step 1 of the analyses, and high-risk patient indicators which were entered in Step 2 (number of GP visits in the past 12 months, recent fall, recent hospitalisation) and Step 3 (polypharmacy, multimorbidity). Missing data were managed by excluding cases listwise. For analysis purposes, nationality was coded as ‘Irish’ or ‘non-Irish’, and ‘GMS’ was collapsed into ‘private patient’ or ‘public patient’. A participant met the criteria for polypharmacy (coded as ‘yes’/‘no’) if they reported being on five or more repeat medications, and were considered multimorbid (‘yes’/‘no’) if they listed the presence of at least two chronic conditions.

#### Open-ended responses of patients

A deductive content analysis approach [26] was used to code the free-text responses to ‘additional comments in relation to safety of care’. These responses were coded according to SC domains explicated by Flin et al. [27] and Madden et al. [15], which included: safety systems; risk perceptions; job demands/personal resources; reporting/speaking up; safety attitudes/behaviours; communication/feedback; teamwork; management; organisational factors; co-ordination of care; staff competence; access and timeliness; facilities and equipment; and dignity and respect. Further, we coded whether each assertation constituted a positive (i.e., good practice) or negative (i.e., poor practice) exemplar [28]. Comments were excluded if they were not considered to convey information about a specific safety domain (e.g., *‘I’m satisfied with my GP’*). Responses were initially coded by the first author (CM), and reviewed by a second author (POC) to enhance trustworthiness. Disagreements were resolved via discussion [29] until consensus was achieved.

## Results

### Participant characteristics

A total of 584 participants completed the survey (57.5% online, 42.5% paper version). There were 500 paper copies administered, yielding a response rate of 49.6% (248/500). We were unable to calculate a response rate for the online version of the survey, as it was not possible to determine the number of contacts each of the support/community groups invited to partake.

Participant demographic characteristics and risk factors are shown in Table 1. Respondents were predominantly female (75.6%), most were Irish (91.3%), and the majority were private patients (72.6%). The mean age was 42.6 years (*SD* = 16.1; range:18–91 years). Participants had attended a mean of 3.60 (*SD* = 3.24; range = 0–30) appointments with a GP in the previous 12 months. The breakdown of additional patient risk factors are detailed in Table 1.

**Table 1:** Participant characteristics

Variable	Frequency^a^ (%)
Gender	
Male	138 (24.4)
Female	427 (75.6)
Nationality	
Irish	533 (91.3)
Non-Irish	36 (6.3)
GMS status	
Medical card	122 (21.4)
GP visit card	34 (6)
Private patient	414 (72.6)
Recent fall	
Yes	45 (7.9)
No	526 (92.1)
Recent hospital admission	
Yes	109 (19.0)
No	464 (81.0)
Polypharmacy	
Yes	69 (12.3)
No	490 (87.7)
Multimorbidity	
Yes	77 (13.4)
No	498 (86.6)

^a^ Numbers do not add to 584 (total *N*), as demographic data were missing for some participants. The valid percent is reported

### Patients’ perceptions of safety climate in Irish general practice

#### Safety climate subscales

Patients reported that they felt safe under the care of their GP, with more than four-fifths of the patients reporting that they either ‘agreed’ or ‘strongly agreed’ with each of the seven items that assessed the ‘Feeling of safety with GP’ subscale. Participants had a median score of 24.0 (*IQR* = 7.0) out of a possible 28 for this subscale.

With a median subscale score of 37.5 (*IQR* = 11.0) out of a possible 48, patients also had favourable perceptions of ‘Practice staff efficiency and teamwork’, with 71.5–90.4% of participants consistently selecting the most positive response options (‘agree’/‘strongly agree’) for each of the 12 items.

In relation to ‘Staff stress and workload’, although over 40% of participants responded that they ‘neither agreed nor disagreed’ that *‘Patient safety is affected by workload in the practice’*, and approximately 30% reported that they were uncertain as to whether ‘*Serious mistakes could happen here’*, participant perceptions remained positive; most ‘strongly disagreed/disagreed’ that the GP was usually stressed, rushed, or distracted (72.7, 56, and 84.9% respectively). Three quarters (75%) of the participants either ‘agreed’/‘strongly agreed’ that the GP had enough time to spend on their care. Participants scored a median of 9.0 (*IQR* = 6.3) out of a possible 28.

In general, participants reported notably positive levels of ‘Patient knowledge and accountability’ (most items > 70%), with the exceptions of *‘The practice answers questions about my care outside of appointments’* and *‘I know how to report issues with my care’*, for which 55.6 and 59% provided positive answers respectively. The mean subscale score was 17.0 (*IQR* = 5.0) out of a possible 24.

Finally, participants had predominantly favourable responses to ‘Safety systems and behaviours’, with over 75–94% consistently agreeing or strongly agreeing to each of the five items, and a median score of 16.0 (*IQR* = 5.0) out of a possible 20.

#### Global safety items

The majority of participants (84.9%) stated that they were either ‘likely’ or ‘extremely likely’ to recommend the practice to friends and family if they needed similar care or treatment. Participants had a median response of 9.0 (*IQR* = 2.0) to ‘on a scale of 0-10, how safe is the care in your practice?’

### Influence of patient risk factors on safety climate perceptions

In the first hierarchical regression, the model was found to account for 3% of the variance in ‘Feeling of safety with the GP’*, F*(9,506) =2.75, *p* = .004. As shown in Table 2, gender (*p* = .04, β = −.09), age (*p* = .002, β = .14) and nationality (*p* = .02, β = .11) were significant predictors, such that being male, of older age, and being Irish were associated with higher subscale scores (i.e., better perceptions of safety).

**Table 2:** Hierarchical multiple regressions of patient risk factors on the PPS-GP subscales

	Feeling of safety with GP			Practice staff efficiency and teamwork			Staff stress and workload			Patient knowledge and accountability			Safety systems and behaviours		
	β	*Adj* R^2^	*F* change	β	*Adj* R^2^	*F* change	β	*Adj* R^2^	*F* change	β	*Adj* R^2^	*F* change	β	*Adj* R^2^	*F* change
**Step 1**		.03	5.44**		.04	6.71**		.03	5.55**		.11	16.44**		.03	4.88
Gender	−.09*			−.06			−.01			−.01			−.10*		
Age	14*			.15**			−.18**			.34**			.11*		
Nationality	.11			.15**			−.08*			.06			.10*		
GMS status	.05			−.00			.02			.01					
**Step 2**		.03	.54		.05	1.45		.03	.74		.10	.19		.03	.98
N visits	.02			.04			−.03			.04			.05		
Recent fall	−.05			−.08			.06			.01			−.07		
Hospitalisation	−.01			.04			.02			.01			−.00		
**Step 3**		.03	.75		.04	.56		.03	.45		.10	.37		.03	1.39
Polypharmacy	−.02			−.02			.03			−.03			−.07		
Multimorbidity	−.05			−.04			.02			−.02			−.07		

*p < .05, **p < .001

In the second regression, the model accounted for 4.4% of the variance in ‘Practice staff efficiency and teamwork’, *F*(9,504) =3.59, *p* < .001. Age and nationality were significant predictors, whereby older age, and being Irish were associated with higher subscale scores.

The third regression accounted for 3% of the variance in ‘Staff stress and workload’, *F*(9,508) =2.80, *p* = .003. Age (*p* < .001, β = −.18) and nationality (*p* = .04, β = .09) were significant predictors, such that older age and being Irish was associated with lower perceived staff stress and workload.

Within the fourth regression, the model accounted for 10.1% of the variance in ‘Patient knowledge and accountability’, *F*(9,503) =7.40, *p* = <.001. Older age (*p* < .001, β = .34) was the only predictor in the model.

Finally, the fifth regression accounted for 3% of the variance in ‘Safety systems and behaviours’, *F*(9,513) =2.80, *p* = .003. Age (*p* = .02, β = −.10) and nationality (*p* = .02, β = .10) were significant predictors, whereby older age, and being Irish were associated with higher subscale scores.

### Open-ended responses of patients

Initially 128 participants provided free-text responses to ‘additional comments in relation to safety’. Of these, 32 were considered ‘neutral’ (e.g. ‘I’m satisfied with my GP’) and excluded from further analysis. Data regarding the frequency of the SC domains identified across the remaining 96 free-text responses, in addition to exemplar quotes related to both good and poor safety practices, are presented in Table 3. A total of 130 codes were identified across the 96 responses, the majority (*n* = 85, 65.4%) of which related to poor SC practices.

**Table 3:** Good and poor practice examples, frequency of emergence data, and exemplar quotes for safety climate domains identified in free-text comment responses

Safety Climate Domain		n (%)^a^	Exemplar quotes
**Communication & feedback**	Good practice	14 (14.6%)	“I was particularly impressed by the communication between nurses and doctors within the practice” “One GP is very good at their job and listens to my concerns”
	Poor practice	15 (15.6%)	“My GP rushed a phone call about scan results and took no time to answer questions” “As a parent, I felt my theory/opinion was ignored and antidepressants were early on for a child”
**Access & timeliness**	Good practice	2 (2.1%)	“They are very efficient with appointment time and the longest I have waited in the waiting room is 10 min” “This [waiting time] doesn’t bother me as they are usually fitting me in”
	Poor practice	17 (17.7%)	“It is difficult to get an appointment. There is usually a wait of at least a week unless it’s an emergency” “My GP couldn’t see me that day to just check it so I had to wait a day to get in, unable to walk”
**Staff competence**	Good practice	8 (8.3%)	“My GP is exceptional…She’s extremely highly skilled and seems to know everything about everything” “My GP was always very knowledgeable”
	Poor practice	13 (13.5%)	“GPs should learn more about ethnic minorities and the health issues that affect them. I usually get misdiagnosed when I go to the GP” “In one situation the GP explained himself that he did not know how to do a speculum exam and referred me to another doctor”
**Dignity & respect**	Good practice	8 (8.3%)	“I’m blessed to finally have found a practice that treats patients with ME/CFS with care, respect, dignity and time” “Doctor, practice nurse and staff are very kind and always treat patients with patience and respect”
	Poor practice	8 (8.3%)	“Feel like a burden, treated with disrespect at times” “The doctors can be a bit condescending sometimes when you’re telling them information you don’t know is relevant or not”
**Job demands**	Good practice	–	–
	Poor practice	16 (16.7%)	“They are extremely busy, and you get the sense it is a stressful environment to work in” “I feel the GPs are so rushed that sometimes they just don’t have sufficient time to spend with acute patients”
**Co-ordination of care**	Good practice	1 (1.0%)	“GP practice is quite new and seems to be well organised”
	Poor practice	9 (9.4%)	“Have to follow up to ensure referrals are made” “We often see different doctors, so part of the appointment is taken up giving the GP your history”
**Risk perceptions**	Good practice	8 (8.3%)	“I would consider my GP practice to be very safe and efficient” “Always felt the safety of care was adequate”
	Poor practice	1 (1.0%)	“If I was a new patient in the practice, I would not feel totally safe”
**Facilities & equipment**	Good practice	1 (1.0%)	“Waiting area is clean and tidy”
	Poor practice	4 (4.2%)	“I’m a wheelchair user, examination bench is not accessible to me. Toilet facilities also not accessible” “No alcohol/disinfectant hand gel anywhere in the waiting/public areas”
**Teamwork**	Good practice	3 (3.1%)	“The staff get along so well, and I receive exceptional treatment at all times from all staff” “The receptionist is a valued member of the medical staff. Just as helpful and always ensures that all messages are sent to the GP.
	Poor practice	1 (1.0%)	“Two GPs in the practice seem to disagree and one has criticised the instructions the other gave me on several occasions”
**Safety systems**	Good practice	–	–
	Poor practice	1 (1.0%)	“There is no ‘red flag’ system, if for example, someone has severe chest pain”

^a^Percentages do not add to 100%, as there were more than one safety climate domains identified across comments. The denominator is 96, the total number of comments

*GP* General Practitioner, *ME/CFS* Myalgic encephalomyelitis/chronic fatigue syndrome

A total of 440 comments relating to areas where changes could be made to GP care were also listed but are not further reported in the current paper as these are the focus of a separate study.

## Discussion

The growing complexity of primary health care delivery presents significant challenges for GPs [4]. Given the important, and under-utilised insights of patients into the care process, the aim of this study was to assess patients’ perceptions of safety climate in Irish general practice, determine whether perceptions differed according to various patient risk factors, and explore whether open-ended responses of patients enhanced our understanding of patient safety information.

Overall, participants reported positive perceptions of safety across all subscales of the PPS-GP. Responses to global safety items were also positive, with participants scoring a median response of 9.0 (*IQR* = 2.0) for overall practice safety rating, on a scale from 0 to 10, and 84.9% ‘likely’ or ‘extremely likely’ to recommend the practice to friends and family. Few other studies have administered patient-report questionnaires to assess safety in the primary care setting [15], which limits our ability to draw extensive comparisons with existing research. Of those in existence, the PREOS-PC conducted in England [30] found that patients had generally positive perceptions of the safety of care provided in general practice, with a mean score of 84.6 out of a potential 100, and 91% agreeing that their HCPs were trustworthy. In a study of patient perceptions of the safety of primary chronic care in Finland [7], 68% either agreed or strongly agreed that they received safe care at home. In a patient-report measure of patient experience of patient-centred medical homes in the US [31], 63% gave positive ratings to their clinic on confidence in quality/safety. It therefore appears that patient perceptions of safety in Irish general practice are more positive than those from other international studies.

The level of favourable views in relation to the ‘Staff stress and workload’ subscale is perhaps surprising, given that a 2015 study of GPs in Ireland reported that 74% rated their stress levels as ‘high’ or ‘very high’ [32]. Further, in a survey of burnout amongst a sample of GPs working in Ireland [33], 52.7% reported high levels of emotional exhaustion, and 6.6% fulfilled the criteria for burnout. However, O’Dea et al. [33] acknowledge that despite high stress levels, Irish GPs continue to derive satisfaction from their work as compared to their international counterparts, and patients are less likely to receive substandard care. This may partly explain why staff workload does not appear to translate to poor patient perceptions of safety. Although this is a relatively positive finding, it is important to also consider the possibility that patients may not actually ‘see’ such system issues. Indeed, a number of qualitative comments which were excluded from the content analysis suggested this (e.g., *‘It’s difficult to answer some of the questions in the survey as its often not possible for me to know. For example, my doctor is stressed- how would I know unless they told me so. They could be extremely stressed but either hiding it well or not even aware if their own stress levels’).* Future research may therefore consider incorporating multiple perspectives of safety climate (e.g., patient and HCP perceptions) in order to obtain an accurate, full picture on systems factors.

Regarding patient risk factors, older age and being of Irish nationality were the only predictors that were significantly associated with positive SC perceptions. Similarly, De Voe et al. [34] report that patients aged over 65 years had positive perceptions of communication with healthcare providers. These patients felt that providers listened to them, showed respect for what they had to say, and spent enough time with them as compared to those aged 18–64. Such perceptions are in spite of findings that older patients are at a greater risk of experiencing a PSI in primary care [35], most commonly related to medication-related incidents, communication-related incidents, and clinical decision-related incidents [36]. Nevertheless, our study demonstrates that older patients feel safer receiving care in Irish general practice- despite their increased risk profile and greater susceptibility to PSIs. We would have also expected participants reporting the presence of certain risk factors such as multimorbidity and polypharmacy would be associated with lower perceptions of safety, given that their complex health profile places them at an increased risk of safety issues [18], and has been associated with a higher occurrence of PSIs [5, 6]; however, poorer SC perceptions were not evident amongst these participants. That non-Irish respondents to our survey felt less safe receiving care than Irish people warrants further exploration to ensure culturally sensitive, safe primary healthcare delivery by targeting language barriers, training needs, and developing guidelines for effective cross-cultural communication [37]; particularly in light of health equity research finding that people of colour are more likely to experience patient safety events [38].

The majority of free-text responses were related to communication and feedback, which is unsurprising given that effective communication has been consistently identified by patients [39, 40] as a key contributor to PSIs. Deficits in relation to access and timeliness were also frequently identified, such as difficulty in obtaining an appointment, which has been cited as a driver of safety problems in other studies [30]. It is, however, surprising that over two-thirds of the responses related to ‘poor’ SC practices, given that the overall SC perceptions were so positive. This finding is similar to research conducted in a hospital setting, whereby patients cited widespread criticism of the hospitalisation experience in response to an open-ended question, despite reporting high satisfaction scores in response to closed-ended questions [41]. The use of an open-ended option in the current study therefore allowed for the exploration of divergent responses to closed-ended questions, and raised issues that would likely have been less noticeable otherwise. This highlights the advantages of using qualitative methods to derive data that provide a deeper and more nuanced understanding [41] of the care experience than collecting quantitative data alone. However, given the disadvantages associated with the use of one format alone (e.g., item non-response to open-ended questions due to time burden [42]) we would suggest that future research combines the use of both qualitative and quantitative methodology when exploring patient-reported safety perspectives. Additionally, these findings emphasise the valuable role that patients can play in identifying poor practices, thus providing information that can be used by GPs to inform safety improvements. This is particularly useful in light of research suggesting reporting that primary care physicians have cited difficulty in understanding how best to measure and improve patient safety in their practices [17].

### Future research

Our finding that patients predominantly identified ‘poor’ SC practices, despite reporting generally positive SC perceptions suggest the need to explore isolated incidents of safety in general practice in greater detail. Previous research has found that even patients with generally positive perceptions of care could recall at least one safety incident they had witnessed previously [43]. Similarly, Ricci-Cabello et al. [30] found that despite participants reporting that providers took adequate measures to ensure safe healthcare delivery, 45% reported experiencing at least one safety problem in the previous 12 months. While the examination of specific incidents was outside the scope of our novel measure, this suggests that despite favourable general perceptions of SC, there may exist a need for future research to further explore the occurrences of isolated incidents of harm.

It has been suggested that ‘a single measure of safety is a fantasy’ [11] and given our findings, the gathering of patient-reported safety information is no exception. There are various purposes, strengths and weaknesses associated with the use of each patient safety measure, which must be considered as complementing each other by providing different levels of qualitative and quantitative information [44]. Therefore, the triangulation of various data collection methods has been recommended to obtain a full view [44, 45] of the safety experience, and ought to be applied to the general practice setting.

Although participants had generally positive perceptions in relation to ‘Patient knowledge and accountability’, just over half reported knowing how to report issues with their care. Similarly, Ricci-Cabello et al. [30] reported low levels of patient activation, with the majority of participants reporting that they ‘never’ or ‘rarely’ raised a concern when they thought something was wrong. In a study of patient complaints, O’Dowd et al. [46] cite a lack of knowledge of the complaints process as a potential reason for patients not complaining [47]. Efforts should be made to ensure that patients are aware of the processes involved in reporting issues with their care, either at a practice or a national level.

### Strengths and limitations

There are a number of limitations that should be considered when interpreting the results of the current study. First, we were unable to calculate a response rate for the online version of the questionnaire, as the recruitment strategy used did not make it possible to collect data on how many patients were invited to complete it. Further, it is reasonable to suggest that it may be lower than the paper version of the survey, given that web response rates have been consistently found to be lower than rates achieved using traditional data collection methods in public health research [48]. Despite this, our response rate is considerably higher than other patient-report safety measures conducted across primary [30] and secondary care [49, 50] settings.

Second, our study sample may not be representative of the population as a whole. Participants self-selected into the study, which may have imparted a self-selection bias, whereby certain types of participants were more likely to participate (e.g., those who were more motivated and more positive [51]). Further, 75% of respondents were female; although Irish data has suggested that women use GP services more frequently than men [52], our figure is disproportionate. Despite contacting a number of chronic illness support groups to share study information, only 14% of participants reported multimorbidity. It would be expected that this would be higher in a representative sample, given that approximately 27% of Irish adults report the presence of at least one long-standing illness or health problem [52]. The majority of respondents were also of Irish nationality, which may be partly explained by the PPS-GP being solely administered in English. Therefore, future research in this area ought to give further consideration to targeted recruitment strategies to capture the safety perceptions of those less well represented populations (i.e., male and multimorbid patients), with a particular emphasis on engaging non-Irish participants (e.g., by translating the measure into different languages), particularly in light of our findings that non-Irish respondents have poorer SC perceptions.

Third, although some significant regression coefficients were observed, suggesting that age and nationality are related to SC perceptions, a small portion of the variance in SC was explained by our set of predictors. This would suggest that our regression models provided poor fit. However, it has been reported that low variance in regression models have been consistently found in previous patient-report studies of healthcare [41] and this is relatively common in social sciences research.

Finally, some, but not all, of the participants were recruited in the midst of the COVID-19 pandemic. Although there is no known research to date specifically on patient-reported perceptions of safety in primary care during COVID-19, as we have acknowledged previously [16], some studies have reported that patient satisfaction has been found to be higher in the COVID-19 period than in the period immediately before [53]. It is therefore possible that there was an artefact from the pandemic (e.g., positive skewness of item responses), given the established links between patient safety perceptions and patient satisfaction [43].

## Conclusion

Our findings indicate that despite being under-utilised, the perceptions of patients are a valuable source of information for measuring safety climate, with promising implications for safety improvement. Further consideration ought to be given on how best to harness these perceptions to allow GPs to access and capitalise on them. Given that our application of a novel, patient-report measure of safety climate yielded deviant responses between quantitative items and an open-response qualitative item, the use of a quantitative survey alone may not adequately capture the entire patient safety experience. We recommend further qualitative exploration of isolated incidents of safety from the patient perspective, and the combined use of qualitative and qualitative methodology to obtain safety information in future research.

## Data Availability

The data that support the findings of this study are available from the corresponding author, CM, on reasonable request.

## Abbreviations

SC: Safety Climate
PPS-GP: Patient Perspective of Safety in General Practice
GP: General Practitioner
PSI: Patient Safety Incident
HCP: Health Care Professional
STROBE: Strengthening the reporting of observational studies in epidemiology
GMS: General Medical Services
DVC: Doctor visit card
SPSS: Statistical Package for the Social Sciences
IQR: Inter-quartile Range
US: United States
PREOS-PC: Patient Reported Experiences and Outcomes of Safety in Primary Care
